# Association Rule Extraction from XML Stream Data for Wireless Sensor Networks

**DOI:** 10.3390/s140712937

**Published:** 2014-07-18

**Authors:** Juryon Paik, Junghyun Nam, Ung Mo Kim, Dongho Won

**Affiliations:** 1 College of Information and Communication Engineering, Sungkyunkwan University, Suwon-si 440-746, Korea; E-Mails: wise96@skku.edu (J.P.); ukim@skku.edu (U.M.K.); 2 Department of Computer Engineering, Konkuk University, 268 Chungwondaero, Chungju, Chungcheongbukdo 380-701, Korea; E-Mail: jhnam@kku.ac.kr

**Keywords:** data mining, wireless sensor network, XML stream data, association rule

## Abstract

With the advances of wireless sensor networks, they yield massive volumes of disparate, dynamic and geographically-distributed and heterogeneous data. The data mining community has attempted to extract knowledge from the huge amount of data that they generate. However, previous mining work in WSNs has focused on supporting simple relational data structures, like one table per network, while there is a need for more complex data structures. This deficiency motivates XML, which is the current *de facto* format for the data exchange and modeling of a wide variety of data sources over the web, to be used in WSNs in order to encourage the interchangeability of heterogeneous types of sensors and systems. However, mining XML data for WSNs has two challenging issues: one is the endless data flow; and the other is the complex tree structure. In this paper, we present several new definitions and techniques related to association rule mining over XML data streams in WSNs. To the best of our knowledge, this work provides the first approach to mining XML stream data that generates frequent tree items without any redundancy.

## 1. Introduction

Wireless sensor networks (WSNs) have been identified as an important research area for the 21st century [1]. The technologies related to WSNs, such as GPS, RFIDs, sensors and *ad hoc* networks, have recently attracted enormous attention in building a smart computing lifestyle. These technologies have been pervasively used in smart and ubiquitous applications, e.g., like healthcare, retail stores, industrial automation, security, disaster protection, academic area and asset management [2]. In such applications, real-time and reliable monitoring is the essential requirement, which is mainly supported by the proliferation of WSNs.

Wide area sensor infrastructures yield massive volumes of dynamic and heterogeneous data flowing through the system [3] and introduce new and unique challenges in the management and control of the data stream. One of the major challenges is extracting useful knowledge about the environment monitored by a WSN system [4]. Extracting useful information from WSN data is commonly called mining stream data and can be done by using typical analysis tools, like association rule extraction, classification and clustering.

Mining stream data differs from mining traditional data in several aspects [5,6]. Firstly, each data element in stream data should be examined, at most, once. This nature of streaming data makes it indispensable to use online algorithms that require only one time scan over the entire data for knowledge discovery. Secondly, memory usage for mining data streams should be bounded regardless of the continuous generation of new data elements. This requirement motivates the design of an in-memory data structure consuming a small amount of memory. Thirdly, each data element in data streams should be processed as fast as possible. Fourthly, the results generated by the online algorithms should be instantly made available to users upon request. Finally, the frequency of errors in the outputs generated by the online algorithms should be constricted to be as small as possible. Due to these differences, previous multiple-pass data mining techniques presented for traditional data sets cannot be directly applied to the domain of mining the stream data.

Previous work for mining stream data has focused on supporting simple relational data structures, like one table per network, while there is a need for more complex data structures. Compared to simple data structures, complex data structures are more suited for efficiently handling large heterogeneous stream data sets. Moreover, the use of a standardized format is desirable for exchanging stream data. A highly interchangeable and extensible data format is XML, which has become the *lingua franca* for exchanging and modeling data from a wide variety of sources over the web. Using XML in WSNs encourages the interchangeability of heterogeneous types of sensors and systems and also makes it easy to interconnect a sensor network to the Internet.

The Sensor Web [7,8] mirrors the idea of sharing, finding and accessing sensors and their data across different applications over a sensor networks and the Internet. The Sensor Web Enablement (SWE) initiative of the Open Geospatial Consortium (OGC) standardizes web service interfaces and data encodings, which can be used as building blocks for a Sensor Web. SWE defines the term Sensor Web as “Web accessible sensor networks and archived sensor data that can be discovered and accessed using standard protocols and application programming interfaces”. When the network connection is accomplished with the Internet and web protocols, XML schemas can be used to issue formal descriptions of the sensor's capabilities, location, interfaces, and so on, which is the framework of XML-based standards. The XML-based data format supports observations and measurements (O&M) to exchange sensor data in an interoperable way, which is becoming increasingly popular. XML provides flexibility and extensibility with an efficient means to package large amounts of data as ASCII or binary blocks.

However, mining XML stream data remains a challenging research area, due to some characteristics of XML stream data. First, XML documents form a tree structure to achieve flexibility, and this makes XML mining more challenging than mining in the traditional, well-structured world. Extracting information from the XML world is still at a nascent stage compared to the fruitful achievements in the relational database community. It is not trivial work to discover useful, but hidden information from a collection of trees [9]. Second, data streams arrive continuously with a high speed and contain a huge amount of data, so that fast processing of the data is very important. In addition, due to the fast data flow, algorithms must scan the data set only once [10].

The main contribution of this paper is to propose a novel and efficient scheme for mining XML stream data. The proposed scheme requires only a one time scan over the streamed XML data. To the best of our knowledge, our proposed scheme is the first approach to mining XML stream data in the sense that it generates frequent tree items without any redundancy (see Section 4 for the definition of a tree item). No redundancy is achieved by employing the label projection technique of Paik *et al*. [11]. To this end, we use a structure consisting of all frequent tree items, called the maximal fraction, as well as structures similar to lists constituting a label projected database. The overall methodology of our scheme can be applied to an individual block, as well as the whole stream. This feature enables our scheme to discover frequent tree items better than the previous schemes.

The rest of this paper is organized as follows: Section 2 discusses prior work related to mining association rules from sensor data and XML data. Section 3 gives some preliminaries on association rules and XML data structures. Then, in Section 4, we describe the problem of mining association rules from XML stream data and provide some definitions with respect to mining XML stream data. Afterwards, Section 5 presents our proposed scheme and compares it with previously published ones. We conclude this paper and suggest some future work in Section 6.

## 2. Related Work

Recently, extracting knowledge from stream data has received great attention by the data mining community [12], because many modern applications require the robust transmission of streaming data over a sensor or telecommunication network. Different approaches focusing on clustering, classification and association rule discovery have been successfully used on stream data. Among them, our aim is to discover association rules.

The problem of mining association rules was first introduced in [13] to analyze customer behaviors in retail databases consisting of traditional relational data. The mined association rules enabled retailers to predict the items that could be purchased together within a single transaction. The use of association rules has a great influence on making decisions about which item should be put on sale or which items should be placed near each other. A large amount of work has been done in various directions. The famous Apriori algorithm for extracting association rules was published independently in [14] and in [15]. Subsequently, many algorithms have been developed with adaptations of different optimization techniques [16–18]. The FP-Growth method of Han *et al.* [18] makes two main improvements over the previous methods. First, it uses the FP-tree data structure, which is a compressed form of the database and, thus, provides memory savings. Furthermore, there is no candidate set generation in FP-Growth, which makes the overall algorithm fast. Our proposed scheme makes use of a similar idea to the one behind FP-Growth.

A framework for discovering association rules from sensor networks was proposed by Loo *et al.* [19]. In Loo *et al.*'s framework, a data model for storing stream data was presented to employ the lossy counting algorithm, which enables online one-pass analyses of data. In [20], Halatchev and Gruenwald proposed a data estimation technique that uncovers meaningful relationships between sensors via stream data mining based on closed frequent itemsets (CARM). The mined relationships between sensors are used to recover missing or damaged sensor data. This recovery feature helps to improve the efficiency of the mining algorithm in terms of both time and space. Boukerche and Samarah [21] proposed a comprehensive framework for mining patterns regarding sensors' behaviors in wireless *ad hoc* sensor networks (WASNs). The new formulation presented by Boukerche and Samarah captures the temporal relations between sensors. Such relations can be used in identifying the correlated sensors, thereby improving the quality of service of WASNs. The fundamental strategy in Boukerche and Samarah's framework is to optimize the number of messages exchanged for a mining sensors' association rules.

So far, only a few studies have attempted to address the problem of extracting association rules from XML stream data for wireless sensor networks (all of the schemes discussed above have focused on mining from simple relational stream data). Recently, Corpinar and Gündem [10] introduced a mining scheme called PNRMXS,which builds upon the FP-Growth method of Han *et al.* [18]. PNRMXS mines both positive and negative association rules on XML data streams by using the correlation coefficient measurement. Our proposed scheme is based on Han *et al.*'s FP-Growth method [18], as well as Paik *et al.*'s XML mining technique [11]. Compared with PNRMXS, our scheme generates and uses maximal frequent tree items without redundancy.

## 3. Preliminaries

This section provides some definitions and background needed to understand association rule mining and the XML data structure.

### 3.1. Association Rules for Relational Data

Let 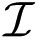 be a set of items: *I_1_*, *I_2_*,…, *I_n_*. An association rule is an implication of the form *X* ⇒ *Y*, where the rule body *X* and head *Y* are subsets of 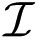, such that *X* ∩ *Y* = *φ*. Let 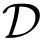 be a set of transactions. Then, a rule *X* ⇒ *Y* states that a transaction *T* ∈ 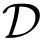 containing the items in *X* (*i.e*., *X* ⊂ *T*) is likely to contain also the items in *Y* (*i.e., Y* ⊂ *T*).

There are two measures that characterize the given association rules: support and confidence. The former measures the percentage of transactions in 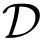 that contain all of the items in *X* and *Y*, and the latter measures the percentage of transactions containing the items in *Y* among the transactions in 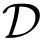 containing the items in *X*. More formally, given the function *freq*(*X*, 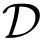), which denotes the percentage of transactions in 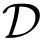 containing *X*, we define:

(1) support(X⇒Y)=freq(X∪Y,D)

and:

(2) $$\text{confidence}(X\Rightarrow Y)=\frac{\text{freq}(X\cup Y,D)}{\text{freq}(X,D)}$$

Suppose there is an association rule *bread*, *butter* ⇒ *milk*, the famous rule provided in [13], with confidence 0.9 and support 0.05. The rule states that customers who buy bread and butter also buy milk in 90% of the cases and that this rule holds for 5% of the transactions. The problem of mining association rules from a set of transactions 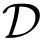 is to generate all of the association rules that have support and confidence greater than two user-given thresholds: minimum support and minimum confidence.

### 3.2. XML Data Structure

XML represents data as trees and makes no requirement that the trees be balanced [22–25]. Indeed, XML is remarkably free-form, with the only requirements being that: (1) the root is the unique node denoting a whole document; (2) the other internal nodes are labeled by tags; and (3) the leaves are labeled by the contents or attributes of tags. A rooted tree is a directed acyclic graph satisfying that: (1) there is a special node called the root that has no entering edges; and (2) every other node has exactly one entering edge. Thus, any XML tree is a rooted tree.

Let *T* = (*r*,*V*, *E*, *L*) denote a tree, where *r* ∈ *V* is the root node, *V* is a set of nodes, *E* is a set of edges and *L* is the set of labels. We say that the tree *T* is a labeled tree if there exists a labeling function 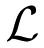 that assigns a label to each node in *V*. For any node *v* ∈ *V*, 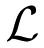
(*v*) ∈ *L* is the label of *v*. The size of a tree *T*, denoted as |*T*|, is defined as the number of nodes the tree has.

A path in a tree is a sequence of edges of the form 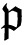 = 〈(*v*_1_, *v*_2_), (*v*_2_, *v*_3_), …, (*v_m_*_−2_, *v_m_*_−1_), (*v_m_*_−1_, *v_m_*)∈, where *v*_1_,…, *v_m_* ∈ *V.* For short, we represent the path 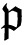 just by the distinct nodes on the path; *i.e*, 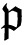 = 〈*v*_1_, *v*_2_, *v*_3_,…, *v_m_*_−1_, *v_m_*〈*.* The length of a path is the number of edges on the path; the length of 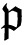 is *m* − 1. There is a unique path from the root to each node in a tree.

#### Definition 1

*If u, v* ∈ *N and there is a path* 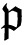 *from u to v, then u is called an ancestor of v, while v is called a descendant of u. If u is an immediate ancestor of v* (*i.e.*, (*u, v*) ∈ 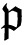), *then u is called the parent of v, while v is called the child of u.*

Every node (except for the root and leaves) has exactly one parent and one or more children. Nodes that share the same parent are siblings. A node with no children is a leaf node; otherwise, it is an internal node.

Tree inclusion is used as a means of retrieving information from trees [26]. Given a pattern tree *S* and a target tree *T*, the general tree inclusion problem is to find the subtrees of *T* that are instances of *S.* In this context, the subtrees of *T* are said to occur or match at the root of the trees that are instances of the pattern tree *S*. The discovery of matching subtrees is not a trivial task, because of the hierarchy characteristics of trees. Several types of related subtree definitions have been given in recent work for tree mining [22,25–27].

#### Definition 2

*Given a tree T* = (r, V, E, L), *we say that an ordered tree S* = (*r′, V_S_, E_S_, L′*) *is included as an exact subtree of T, denoted S* ⪯ *T, iff: (1) V_S_* ⊆ *V; (2) E_S_* ⊆ *E; (3) for a node v* ∈ *V, if v* ∈ *V_S_, then all descendants of v must be in V_S_; (4) for all edges (u, v)* ∈ *E_S_, the parent-child relation between node u and v is preserved in T identically with the one in S; (5) for any node v* ∈ *V_S_*, 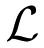
(*v*) ∈ *L′* Λ 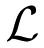
(*v*) ∈ *L; and* (*6*) *the left to right ordering between the siblings in S must be preserved in T.*

#### Definition 3

*Given a tree T* = (*r, V, E, L*), *we say that an unordered or ordered tree S* = (*r′, V_S_, E_S_, L′*) *is included as an embedded subtree of T, denoted S ⪷) T, iff: (1; V_S_* ⊆ *V; (2) for all edges (u, v)* ∈ *E_S_, such that u is the parent of v, u is an ancestor of v in T; and (3) for any node v* ∈ *V_S_*, 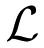
(*v*) ∈ *L′* Λ 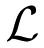
(*v*) ∈ *L.*

Throughout the paper, we focus on embedded subtrees from the dataset of XML stream data and use them in providing the definitions for association rules.

## 4. A Framework for XML Stream Data Mining

Due to its flexibility and easy interchangeability, XML is used as the standard format for transmitting stream data generated by sensors in an increasing number of WSN applications. This section presents a new framework for mining association rules from XML stream data. We make the following assumptions on stream data.

- The size of each block of the data stream is identical; each block contains the same number of transactions.
- Sink nodes collect their data from sensor nodes, and therefore, the target data sets to be used in our mining are obtained from the sink nodes.

### 4.1. Item Sets

In traditional association rule mining, the basic unit of data is a database record, and the construction unit of a discovered association rule is an item with an atomic value [28]. This subsection aims to define the XML counterparts of record and item. Our definitions can be seen as combined variants of the definitions from traditional domains [2,3,12] and XML domains [11,28].

**Figure 1.**
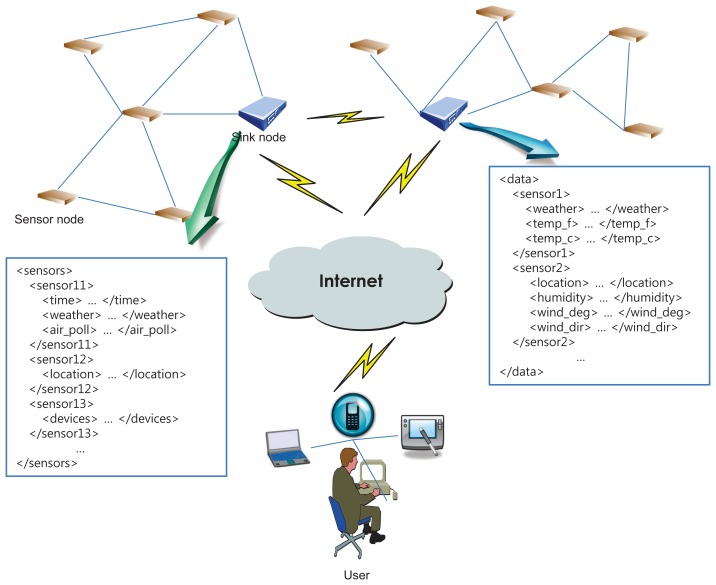
A system architecture for a WSN environment.

Figure 1 depicts a system architecture for a WSN environment [2,29,30] and presents simple examples of XML-encoded sensor data. In the figure, the whole network is a configuration of two subnetworks, which differ in their sensing area, integrated with the Internet. In each subnetwork, the sink node with relatively sufficient resources serves as a control center for gathering required information. Usually, sensing data are stored in sensor nodes when an event is detected. Then, the sink node travels in its sensing area and collects data from the sensors.

Since we focus on the rule detection from XML stream data, each XML document corresponds to a set of XML data in a sink node, and the data stream is a continuous sequence of XML data blocks. As mentioned above, we assume that each block contains the same number of transactions. We now proceed to define what a transaction exactly means in the context of XML stream data mining.

Let XML data stream *XDS* = (*XB*_1_, *XB*_2_,…, *XB*_∞_) be a sequence of XML blocks, where the identifier *XB*_∞_ is the latest block. Each block *XB_i_*, 1 ≤ *i* ≤ ∞ consists of a timestamp *t_i_* and a set of transactions; that is, *XB_i_* = (*t_i_*, {*T*_1_, *T*_2_,…, *T_n_*}), where *n* > 0. Therefore, the length of the data stream depends on a total number of transactions arriving until the latest timestamp, *t*_∞_.

#### Definition 4

*Given an XML data stream XDS* = (*XB*_1_, *XB*_2_,…, *XB*_∞_), *the size of a block XB_i_ is denoted as* |*XB*_i_| *and is defined as the number of its transactions. Then, the length of an XML data stream is defined as* $\left|\text{XDS}\right|=\sum_{i=1}^{\infty }\left|XB_{i}\right|=\left|XB_{1}\right|+\left|XB_{2}\right|+\ldots +\left|XB_{\infty }\right|$.

Every transaction *T_j_* in each block *XB_i_* is an XML document and, thus, has a structure of a rooted labeled tree. Since any portion of a tree also has a tree structure, any part of a transaction can potentially become an item. We name this possible item a fraction. We say that a tree *F* = (*r_F_*, *v_f_, e_f_, L_F_*) is included as an embedded fraction of a tree *T*, denoted as *F* ⪷ *T*, if *F* and *T* satisfy the conditions of Definition 3. Intuitively speaking, the fraction *F* must not break the ancestor-descendant relationships between the nodes in the tree *T.*

We call a fraction used in an association rule a tree item, titem for short, to differentiate it from an item defined for relational data. Any fraction is eligible to be a titem, because the whole XML document consists of several fractions, and the structure of a fraction is also a tree.

### 4.2. Association Rules

Based on the notions of transaction, fraction and titem, we now formally define an association rule and some related measurements for XML stream data. For the given XML data stream *XDS* = (*XB*_1_, *XB*_2_,…, X*B*_∞_), the rule measuring process is done over each individual block *XB_i_*. Assume again that *XB_i_* = (*t_i_*, {*T*_1_, *T*_2_…*T_n_*}). Let 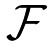 = {*F_jk_, k* > 0 | *F_jk_* ⪯ *T_j_*,0 < *j* ≤ *n*} be a total set of fractions collected from all blocks and 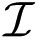 = {*I*_1_, *I*_2_ … *I_m_*} be a set of titems. Then, *T_j_* ⊆ 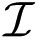 ⊆ 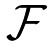. Any transaction has its unique identifier, called the transaction identifier, and we denote it by the subscript *j*.

Let *X* = {*x*_1_, *x*_2_,…, *x_f_*} and *Y* = {*y*_1_, *y*_2_,…, *y_g_*} be two titem sets, such that *X, Y* ⊂ 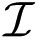. An XML stream data association rule is the implication of the form *X* ⇒ *Y* that satisfies the following two conditions: (1) *X* ∪ *Y* ⊂ 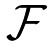; and (2) *X* ∩ *Y* = ∅.

Each titem set has an associated statistical measurement, named the frequency, abbreviated freq. The frequency of a titem set *X* is denoted as *freq*(*X*) and is generally defined as the number of transactions in which the titem set occurs as a subset [9,28]. For our purposes, we redefine this measurement with two slightly different versions, depending on the target data set.

#### Definition 5

*A titem set X* ⊂ 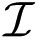 *has two types of frequencies: one is a block-frequency, abbreviated bfreq, and the other is a stream-frequency (sfreq). (1) A block-frequency of X, bfreq(X), is the number of transactions in any given block. For instance, if the given block is XB_p_, then* $\text{bfreq}(X,XB_{p})=\left|XB_{p}^{X}\right|=\left|\{T_{j}\right|\left(X\subseteq T_{j}\right)\wedge \left(T_{j}\in XB_{p}\right)$, *for p* ∈ [1,∞], *j* > 0}|. (2) A stream-frequency of X, sfreq (X), is the total number of transactions in a given XML data stream XDS. That is, $\text{sfreq}(X,\text{XDS})=\left|XDS^{X}\right|=\sum_{i=1}^{\infty }\left|XB_{i}^{X}\right|=\left|XB_{1}^{X}\right|+\left|XB_{2}^{X}\right|+\ldots +\left|XB_{\infty }^{X}\right|=\left|\{T_{j1}\right|\left(X\subseteq T_{j1})\wedge (T_{j1}\in XB_{1}\right)|+\left|\{T_{j2}\right|\left(X\subseteq T_{j2})\wedge (T_{j2}\in XB_{2}\right)|+\ldots +\left|\{T_{jn}\right|\left(X\subseteq T_{jn})\wedge (T_{jn}\in XB_{\infty }\right)|$.

A given titem set *X* is called a *XB_p_*-frequent titem set with respect to the block *XB_p_* if *bfreq*(*X, XB_p_*) ≥ *δ_b_* × |*XB_p_*|, where *δ_b_* is a user-specified threshold for the block *XB_p_* and 0 ≤ *δ_b_* ≤ 1. Otherwise, it is *XB_p_*-infrequent. Similarly, *X* is called frequent if *sfreq*(*X,XDS*) ≥ *δ_s_* × |*XDS*|, where *δ_s_* is the threshold for the stream data and 0 ≤ *δ_s_* ≤ 1. Otherwise, *X* is infrequent for the stream data.

### 4.3. Support and Confidence

The strength and reliability of an association rule *X* ⇒ *Y* can be measured in terms of its support and confidence. Support determines how often a rule is applicable to a given data set, while confidence determines how frequently the titem set *Y* appears in transactions that contain the titem set *X*. The formal definitions of these metrics over an XML stream data set are given in two aspects: Over each block and the whole stream data. The Equations (1) and (2) should be adjusted to cover the XML stream data.

#### Definition 6

*Given XSD, the support and confidence of an association rule X* ⇒ *Y are defined in two ways: block and stream. Accordingly, there are four ways of measuring strength and reliability: block-support, block-confidence, stream-support and stream-confidence.*

- *The block-support and block-confidence of rule X* ⇒ *Y in any given block XB_p_ are denoted as* bsup(*X* ⇒ *Y*, *XB_p_*) *and* bconf(*X* ⇒ *Y, XB_p_*), *respectively, and are defined as*:
  - $\text{bsup}(\text{X}\Rightarrow Y,\text{X}B_{\text{p}})=\frac{\text{bfreq}(\text{X}\cup Y,XB_{p})}{\left|XB_{p}\right|}=\frac{\left|XB_{p}^{X\cup Y}\right|}{\left|XB_{p}\right|}$,
  - $\text{bconf}(\text{X}\Rightarrow \text{Y},{\text{XB}}_{\text{p}})=\frac{\text{bsup}(\text{X}\cup \text{Y},{\text{XB}}_{\text{p}})}{\text{bsup}(\text{X},{\text{XB}}_{\text{p}})}=\frac{\text{bfreq}(\text{X}\cup \text{Y},{\text{XB}}_{\text{p}})}{\text{bfreq}(\text{X},{\text{XB}}_{\text{p}})}=\frac{\left|XB_{p}^{X\cup Y}\right|}{\left|XB_{p}^{X}\right|}$.
- *The stream-support and stream-confidence of rule X* ⇒ *Y in the whole XML stream data are denoted as ssup*(*X* ⇒ *Y, XDS*) *and sconf*(*X* ⇒ *Y, XDS*), *respectively, and are defined as*:
  - $\text{ssup}(\text{X}\Rightarrow \text{Y},\text{XDS})=\frac{\text{sfreq}(X\cup Y,\text{XDS})}{\left|\text{XDS}\right|}=\frac{\left|XDS^{X\cup Y}\right|}{\left|\text{XDS}\right|}$,
  - $\text{sconf}(\text{X}\Rightarrow Y,\text{XDS})=\frac{\text{ssup}(\text{X}\cup Y,\text{XDS})}{\text{ssup}(X,\text{XDS})}=\frac{\text{sfreq}(X\cup Y,\text{XDS})}{\text{sfreq}(X,\text{XDS})}=\frac{\left|XDS^{X\cup Y}\right|}{\left|XDS^{X}\right|}$.

A rule discovery procedure is to find association rules of the form *X* ⇒ *Y* having their supports and confidences greater than or equal to the user-specified minimum support and minimum confidence, denoted as *ms* and *mc*, respectively. We use *bms* and *bmc* to denote *ms* and *mc* given for a block, and use *sms* and *smc* to denote *ms* and *mc* given for the whole stream.

**Figure 2.**
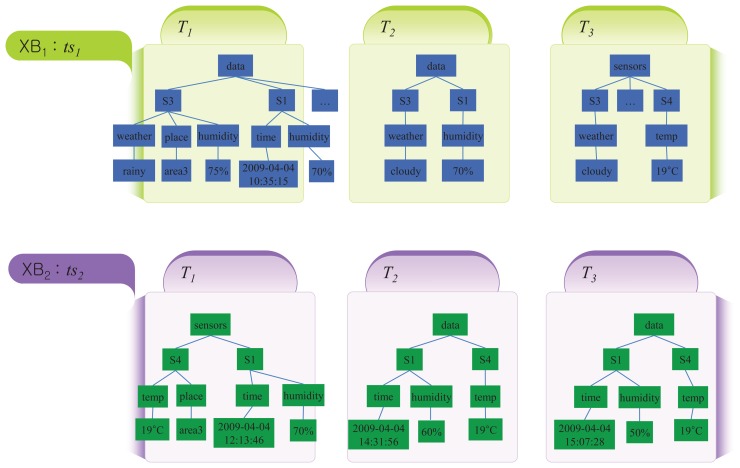
An example of XML stream data with two blocks, each having three transactions.

Let us consider the XML stream data shown in Figure 2, where several sensor nodes provide various information to their sink nodes. We assume that the XML stream data contains two blocks, *i.e*., *XSD* = {*XB*_1_, *XB*_2_}, and *XB*_2_ is the latest block with timestamp *ts*_2_. The size of each block is three, meaning both blocks have three transactions (trees). That is, |*XB*_1_| = |*XB*_2_| = 3 and |*XSD*| = 6. The transactions deliver information, like weather, humidity, temperature, and so on.

To make the fractions that encompass all possible titems, we start from the fractions with one node and then extend those fractions to the bigger ones by adding nodes one by one. A detailed description of this process will be given in Section 5.

**Figure 3.**
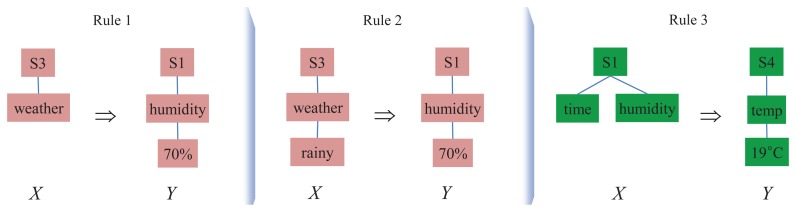
Association rule candidates configured with titems from the fractions of *XSD* in Figure 2.

In Figure 3, we consider three different candidate rules derived from the fractions of the stream data *XSD* in Figure 2. Each of the candidates is formed by two titems selected from the fractions of *XSD*. We first measure the frequencies of each candidate rule as per Definition 5.

1. Rule 1:
  (a) $b\text{freq}(X,XB_{1})=\left|XB_{1}^{X}\right|=3$, $b\text{freq}(Y,XB_{1})=\left|XB_{1}^{Y}\right|=2$
  (b) $b\text{freq}(X,XB_{2})=\left|XB_{2}^{X}\right|=0$, $b\text{freq}(Y,XB_{2})=\left|XB_{2}^{Y}\right|=1$
  (c) sfreq(X,XDS)=3+0=3, sfreq(Y,XDS)=2+1=3
2. Rule 2:
  (a) $b\text{freq}(X,XB_{1})=\left|XB_{1}^{X}\right|=1$, $b\text{freq}(Y,XB_{1})=\left|XB_{1}^{Y}\right|=2$
  (b) $b\text{freq}(X,XB_{2})=\left|XB_{2}^{X}\right|=0$, $b\text{freq}(Y,XB_{2})=\left|XB_{2}^{Y}\right|=1$
  (c) sfreq(X,XDS)=1+0=1, sfreq(Y,XDS)=2+1=3
3. Rule 3:
  (a) $b\text{freq}(X,XB_{1})=\left|XB_{1}^{X}\right|=1$, $b\text{freq}(Y,XB_{1})=\left|XB_{1}^{Y}\right|=1$
  (b) $b\text{freq}(X,XB_{2})=\left|XB_{2}^{X}\right|=3$, $b\text{freq}(Y,XB_{2})=\left|XB_{2}^{Y}\right|=3$
  (c) sfreq(X,XDS)=1+3=4, sfreq(Y,XDS)=1+3=4

Using the calculated frequencies, the supports and confidences of each rule are measured according to Definition 6.

1. Rule 1:
  (a) $\text{bsup}(X\Rightarrow Y,XB_{1})=\frac{\left|XB_{1}^{X\cup Y}\right|}{\left|XB_{1}\right|}=\frac{2}{3}≃0.67$
  (b) $\text{bsup}(X\Rightarrow Y,XB_{2})=\frac{\left|XB_{2}^{X\cup Y}\right|}{\left|XB_{2}\right|}=\frac{0}{3}=0.0$
  (c) $\text{ssup}(X\Rightarrow Y,\text{XDS})=\frac{\left|XDS^{X\cup Y}\right|}{\left|\text{XDS}\right|}=\frac{2}{6}≃0.33$
2. Rule 2:
  (a) $\text{bsup}(X\Rightarrow Y,XB_{1})=\frac{\left|XB_{1}^{X\cup Y}\right|}{\left|XB_{1}\right|}=\frac{1}{3}≃0.33$
  (b) $\text{bsup}(X\Rightarrow Y,XB_{2})=\frac{\left|XB_{2}^{X\cup Y}\right|}{\left|XB_{2}\right|}=\frac{0}{3}=0.0$
  (c) $\text{ssup}(X\Rightarrow Y,\text{XDS})=\frac{\left|XDS^{X\cup Y}\right|}{\left|\text{XDS}\right|}=\frac{1}{6}≃0.17$
3. Rule 3:
  (a) $\text{bsup}(X\Rightarrow Y,XB_{1})=\frac{\left|XB_{1}^{X\cup Y}\right|}{\left|XB_{1}\right|}=\frac{0}{3}=0$
  (b) $\text{bsup}(X\Rightarrow Y,XB_{2})=\frac{\left|XB_{2}^{X\cup Y}\right|}{\left|XB_{2}\right|}=\frac{3}{3}=1$
  (c) $\text{ssup}(X\Rightarrow Y,\text{XDS})=\frac{\left|XDS^{X\cup Y}\right|}{\left|\text{XDS}\right|}=\frac{3}{6}=0.5$

Assume that *bms* = *sms* = 0.3. Then, due to the given thresholds, some candidate rules are pruned from the pool of frequent association rules. In the case of block-support, Rules 1 and 2 do not satisfy the *bms* threshold in *XB*_2_, because both are zero. This means that titems *X* and *Y* never occur together in any transaction *T_i_* of *XB*_2_*.* However, both rules are eligible to be frequent association rules in *XB*_1_*.* We say that Rules 1 and 2 are *XB*_1_-support. Rule 3 shows a different result. *X* and *Y* of Rule 3 never occur together within *XB*_1_, but they occur together 100% within *XB*_2_. Thus, Rule 3 is *XB*_2_-support. This result implies that some association rules hold important information for some blocks, but not for other blocks.

Every rule satisfies any one of the block-supports in the example. In the case of stream-support, Rules 1 and 3 are interesting association rules to be extracted, whereas Rule 2 cannot be an association rule, because its support is 0.17 less than the threshold, 0.3.

For the association rules found to be interesting, their reliability should be measured based on the confidence. For a given rule *X* ⇒ *Y*, the higher the confidence, the more likely it is for *Y* to be present in transactions that contain *X*. Confidence also provides an estimate of the conditional probability of *Y* given *X*. *bconf* and *sconf* are computed for the selected association rules, as shown in Definition 6. Then, the resulting values are compared with the given thresholds, *bmc* and *smc*: *bmc* for *XB*_1_-support and *XB*_2_-support and *smc* for Rule 1 and Rule 3. We assume *bmc* = *smc* = 0.3.

1. *XB*_1_-support:
  (a) $\text{bconf}(\text{Rule}1,XB_{1})=\text{bconf}(X\Rightarrow Y,XB_{1})=\frac{\left|XB_{1}^{X\cup Y}\right|}{\left|XB_{1}^{X}\right|}=\frac{2}{3}≃0.67$
  (b) $\text{bconf}(\text{Rule}2,XB_{1})=\text{bconf}(X\Rightarrow Y,XB_{1})=\frac{\left|XB_{1}^{X\cup Y}\right|}{\left|XB_{1}^{X}\right|}=\frac{1}{1}=1$
2. *XB*_2_-support:
  (a) $\text{bconf}(\text{Rule}3,XB_{2})=\text{bconf}(X\Rightarrow Y,XB_{2})=\frac{\left|XB_{2}^{X\cup Y}\right|}{\left|XB_{2}^{X}\right|}=\frac{3}{3}=1$
3. *stream*-support:
  (a) $\text{sconf}(\text{Rule}1,\text{XDS})=\text{sconf}(X\Rightarrow Y,\text{XDS})=\frac{\left|XDS^{X\cup Y}\right|}{\left|XDS^{X}\right|}=\frac{2}{3}≃0.67$
  (b) $\text{sconf}(\text{Rule}3,\text{XDS})=\text{sconf}(X\Rightarrow Y,\text{XDS})=\frac{\left|XDS^{X\cup Y}\right|}{\left|XDS^{X}\right|}=\frac{3}{4}=0.75$

The resulting values of support and confidence enable us to extract various interesting rules, including the following:

- With 100%, Sensor 1 senses “the humidity is 70%” whenever Sensor 3 detects “the weather is rainy”.
- With 75%, Sensor 4 senses “the temperature is 19°C” if Sensor 1 detects both time and humidity

Moreover, based on the stream support and confidence, we can decide that Rule 3 has more strength and reliability than Rule 1.

## 5. Mining XML Stream Association Rules with the Label Projection Approach

The label projection technique, originally presented in [11], turned out to be very useful in reducing the computation complexities of mining algorithms, as it enables one to avoid the generation of uninteresting subtrees and to expedite the extraction of desired subtrees. The label projection technique uses a set of lists to store all necessary information of the tree database, such as the label, node id, tree id and parent/ancestor relationships. Our proposed scheme adapts the label projection technique to make it work for XML stream data. We here provide a brief overview of notions for label projection. Readers are referred to [11,31] for more details.

### 5.1. Scheme and Construction of Label Projection

Like tree or transaction indexes, labels can be used as primary keys in XML stream databases. This means that the trees, actually transactions, in *XSD* can be reorganized according to labels. During the scan of trees, all nodes with the same labels are grouped together spontaneously. In a label-driven layout, the time complexity to check label frequencies requires at most *O*(|*L*||*XSD*|). If a hash-based search is used, the complexity is reduced up to *O*(|*XSD*|).

#### Definition 7

*Let ℓ be a label in some label set L. During the scan of arriving trees, tree indexes and node indexes are projected by the label ℓ and construct a single linked list, called a label list. The label list for the label (x02113; is denoted as (x02113;-list*.

The structure of a label list is similar to that of a linked list in that it has a head and a body. The head of a label list points to the first object of the body, just like the ordinary head of a linked list. The head gives information about which node indexes have been mapped to a projected label. The body is formed in a way that can easily determine: (1) the number of trees having the key of the head; and (2) the parent positions of the nodes in the head; the former is for dealing with the frequency of each label, while the latter is for handling the hierarchical information of the label. To this end, the body is structured as a sequence of members, with each member being an object consisting of a key field, one link field pointing to the next member and one satellite data field.

A tree index number is used as a key, and this means that the label of the head has been assigned to the nodes in the corresponding tree. During the database scan, members are generated and inserted into the bodies of label lists. A newly inserted member is added to the end of an appropriate body and is pointed to by the link field of its previous member. The number of members in a body is called the size of the corresponding label list.

**Figure 4.**
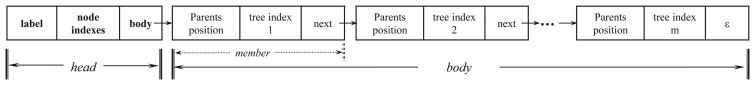
Structure of a label list.

The complete structure of a label list is depicted in Figure 4. As shown in the figure, m trees constitute the *ℓ*-list. Tree indexes are placed in key fields, and parent indexes of the nodes are stored in satellite data fields. Let *T*_1_, *T*_2_ and *T*_3_ be three different trees in *XSD.* Assume that one node is labeled by *ℓ* in *T*_1_ and *T*_3_ and two nodes are labeled by *ℓ* in *T*_2_. Then, *ℓ*-list is 〈(*p*_1_, *T*_1_, →), (*p*_2_*p*_3_,*T*_2_, →), (*p*_4_, *T*_3_, *ε*)∈, where *p_i_* is a parent node index, → means a pointer to the next member and *ε* means an empty pointer. The size of *ℓ*-list, |*ℓ*-list|, is three, because the list body has three members.

The generated label lists are stored and arranged into an in-memory data structure according to the hashed values of the projected labels. Whenever a label is given, its corresponding list is searched and retrieved from the structure to provide the required information. If a label has no matching label list, it is considered a new projected label, and thus, its label list is inserted into the structure. Since the structure works just like an ordinary dictionary, it is called a label dictionary, which we denote as 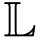
*_dic_*. The size of 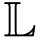
*_dic_*, | 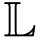
*_dic_*|, is the number of label lists in it.

**Figure 5.**
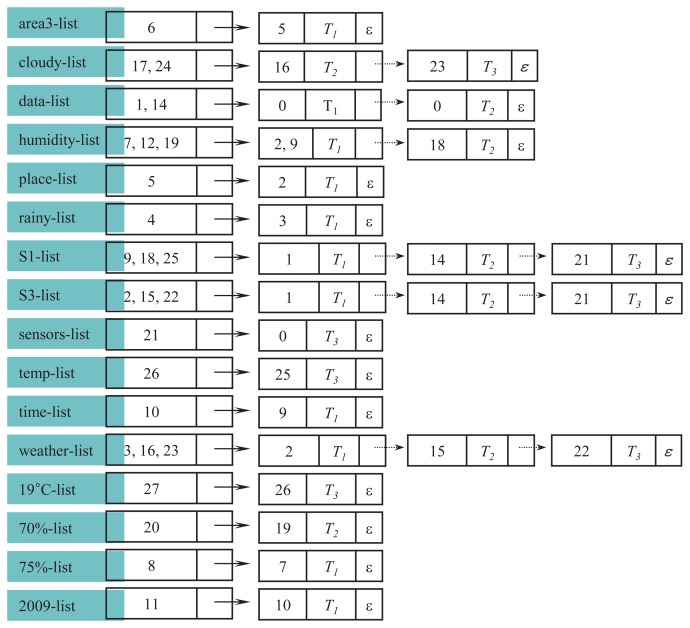
Assembling label lists into 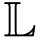
*_dic_.*

Figure 5 shows an example of how 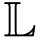
*_dic_* and its units are constructed from the original data set *XSD.* For simplicity, we assume that the entire stream data consists of a single block, *XB*_1_, shown in Figure 2. Hence, we only consider the thresholds for the whole stream, but not for blocks.

In the figure, each number is the node index and *T*_1_,*T*_2_,*T*_3_ are tree indexes. The node whose index is zero represents the root node. The symbol *ε* indicates that there is no next member. Each label ℓ ∈ *L*, where *L* = {*area*3, *cloudy, data, humidity, place, rainy, S*1, *S*3, *sensors, temp,time, weather*, 19*C*, 70%, 75%, 2009}, is projected to generate their label lists. The maximum number of members that can be added into the body of the label list is three, because the total number of trees in *XSD* is three. Thus, the expected size of any label list is between one and three. Then, each label list *ℓ* is stored in 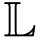
*_dic_* according to the order of their hashed values, 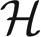
(*ℓ*)*.*

### Pruning and Deriving from 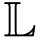
*_dic_*

5.2.

Initially, 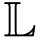
*_dic_* consists of several label lists identified by their unique node label. Some label lists may have labels that do not satisfy the user-given frequency or minimum support. Accordingly, configuring titems with those label lists can produce the rules that do not satisfy the thresholds. Such label lists must not be used in forming association rules. Label lists are filtered out first by their frequency. Recall that *δ_s_* denotes the user-specified stream frequency. If the frequency of a label list is less than the minimum frequency *σ*, which is computed as *σ* = *δ_s_* × |*XSD*|, then the label list should be excluded from 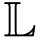
*_dic_.* In the example of Figure 5, *σ* = 2.

#### Definition 8

*An ℓ-list is said to be a frequent label list iff it satisfies the following: (1;*|*ℓ-list* | ≥ *σ; (2) for each parent index p in the members of ℓ-list, the label of p, L(p), has been projected and has L(p)-list; and (3;* |*L(p)-list*| ≥ *σ.*

The label of a parent node *p* has to be frequent in order for an extended subtree to be qualified as being frequent. However, this is not guaranteed in 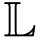
*_dic_*, because filtering was performed only on the frequencies of labels. Therefore, even if parent nodes are included in the label list having a frequent head, it is not certain whether the labels of those parent nodes belong to a label list having a frequent label. This issue can be addressed by modifying the index of every parent node *p* in 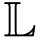
*_dic_*, as shown in the following steps:

1. A parent node in any member is verified by the candidate_hash_table (This table is constructed with the label lists excluded from 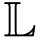
*_dic_*) to check whether the node is assigned an infrequent label or not.
2. If the parent node is assigned an infrequent label, the node is marked ‘replace’, and its record is retrieved from the candidate_hash_table to search a node id assigned a frequent node label.
3. Steps 1 and 2 continue until any node id assigned with a frequent node label is found.
4. The original parent node id is replaced with the found node id, which represents an ancestor node of the original parent node.
5. If no node is found to be frequent, the original parent node id is replaced by zero.

**Figure 6.**
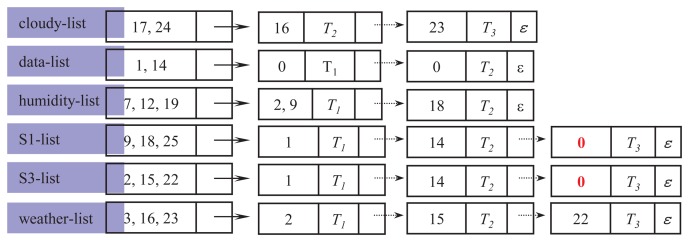
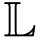
*_dic_* after pruning infrequent label lists.

The result of the pruning phase over the dictionary 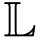
*_dic_* of Figure 5 is presented in Figure 6. As shown in the figure, only six label lists remain, and the rest are pruned, since *σ* = 2. Note that in both S1-list and S3-list, the parent index of their respective third member has been replaced by zero, meaning the root. The volume of data has been dramatically reduced from 100% to 37.5%, approximately, due to the frequent label lists.

Finally, 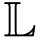
*_dic_* contains all frequent labels and all possibly-frequent paths from root to leaves. The paths in 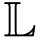
*_dic_* may not be frequent, because: (1) an edge is frequent only if both of its nodes have frequent labels; and (2) a path can be frequent only when all of its edges are frequent. A path 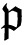 with *m* edges, 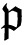 = e_1_e_2_ … *e_m_*, can be represented with a sequence of labels, as shown below:

(3) $$\begin{array}{ll} \text{p} & =e_{1}e_{2}\ldots e_{m}=(v_{1},v_{2})(v_{2},v_{3})\ldots (v_{m},v_{m+1})=(L(v_{1}),L(v_{2}))\ldots (L(v_{m}),L(v_{m+1}))\\ & =L(v_{1})\cdot L(v_{2})\ldots L(v_{m})\cdot L(v_{m+1}) \end{array}$$

In order for 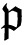 to be frequent, all of the *m* + 1 labels should be frequent labels. The frequency of an edge inside a label list can be verified simply by using the parent index stored in the body part together with the node index in the head part. Finding all frequent edges from 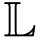
*_dic_* will yield the maximum size of the interesting part of *XSD* that contains all possible titems for configuring association rules.

A fraction is called frequent if its frequency is greater than or equal to *δ_s_*, specified by users or applications. Fractions form a pool from which every titem is selected. The problem of extracting all frequent fractions is to uncover a set *S* of all pattern trees that satisfies the condition *sfreq(S)* ≥ *δ_s_.* However, the combinatorial time for fraction generation becomes an inherent bottleneck of frequent fraction mining, making the problem of finding all frequent fractions harder.

#### Definition 9

*Given some minimum frequency δ_s_, a fraction F is called a maximal frequent fraction with respect to XSD iff it satisfies the following conditions:*

1. *the frequency of F is not less than δ_s_* × |*XSD*|, *i.e.*, sfreq(*F, XSD*) ≥ *δ_s_* × |*XSD*|*.*
2. *there exists no other frequent fraction F′, such that* sfreq(*F′, XSD*) ≥ *δ_s_* × |*XSD*| *and F is a subfraction of F′.*

Simply speaking, a maximal frequent fraction is a frequent fraction that has no frequent, proper, super fraction. Hence, there are fewer maximal frequent fractions compared to the total number of frequent fractions. Despite the fewer total number, maximal frequent fractions do not lose frequent fractions, since they subsume all of them [32,33]. The goal of our scheme is to extract the entire set of maximal frequent fractions from 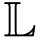
*_dic_*.

Finding maximal frequent fractions starts with determining the symbolic nodes. A symbolic node means a node generated with a label that serves as the key of a label list in 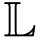
*_dic_*.

#### Definition 10

*Assume a label list ℓ-list. Let p be a parent index in a member of (x02113;-list. A symbolic node s_ℓ_, whose label is ℓ, is set first, and then, the second symbolic node s_L(p)_ with label L(p) is set. These two symbolic nodes are joined together to form an edge. This process is called a label list extension operation, abbreviated ℓ*^2^*e, as the L(p)-list is extended by edges connecting two symbolic nodes. The operation ℓ*^2^*e is denoted as s_L(p)_* → *s_ℓ_, where* → *indicates the direction of extending, parent to child. L(p)* → *ℓ can be interchangeably used with s_L(p)_* → *s_ℓ_.*

The extension process should be done for every label list in 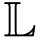
_dic_. Using the label list extension, Equation (3) can be rewritten as follows:

(4) $$\begin{array}{ll} \text{p} & =e_{1}e_{2}\ldots e_{m}=L(v_{1})\to L(v_{2})\to \ldots L(v_{m})\to L(v_{m+1})\\ & =s_{L}(v_{1})\to s_{L}(v_{2})\to \ldots s_{L}(v_{m})\to s_{L}(v_{m+1}) \end{array}$$

Performing the label extension over the entire label lists in 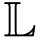
*_dic_* produces a single fraction, where each edge has its own count to monitor how often it occurs in the derived fraction. This phase of building the fraction is supported by the tree_header_table, which stores information, like labels, their locations and flags.

**Figure 7.**
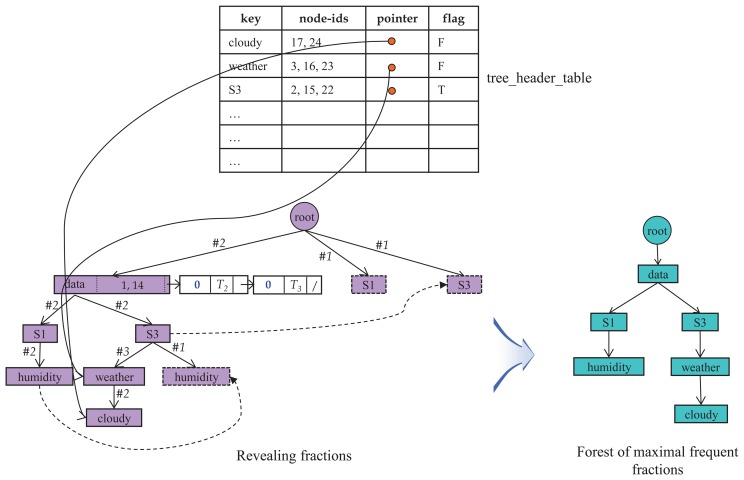
An example of the label extension operation.

Any edge whose count value is less than two is deleted from the maximal fraction. Deleting such edges and rearranging the fraction immediately yield the final outcome, a forest of maximal frequent fractions. Figure 7 shows the final result for the case of our example. In the maximal frequent fraction in the figure, the node labeled ‘root’ is the dummy root and the actual root is the node labeled ‘data’. Every node, edge and path within this fraction are eligible to be titems, and association rules are made from the titems.

Before deriving the forest of maximal fractions, we can infer the number of maximal frequent fractions from the label lists of the final 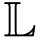
*_dic_*; the number of maximal frequent fractions is the number of label lists that contain *σ* or more members whose index is zero.

Let *ℓ*_1_-list, *ℓ*_2_-list and *ℓ*_3_-list be three arbitrary, frequent label lists in 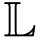
*_dic_.* Assume that |*ℓ*_1_-list| = |*ℓ*_2_-list| = 2, |*ℓ*_3_-list| = 4 and *σ* = 2. For simplicity, we assume that each member of the lists has only one parent index, and all members of *ℓ*_1_-list and *ℓ*_2_-list have the parent node index zero. Then, *ℓ*_1_-list is 〈(0,*T*_1_,→), (0, *T*_2_, *ε*)∈ and *ℓ*_2_-list is 〈(0, *T*_1_,→), (0, *T*_2_, *ε*)∈, meaning that there may be two maximal frequent fractions. Let *p*_1_, *p*_2_, *p*_3_ and *p*_4_ be the parent node indexes of the members of *ℓ*_3_-list. Then, we can consider the following three cases:

- Case 1: *L*(*p*_1_) = *L*(*p*_2_) = *ℓ*_1_ and *L*(*p*_3_) = *L*(*p*_4_) = *ℓ*_2_. Two nodes labeled by *ℓ*_1_ and *ℓ*_2_ are the direct children of the root, because both edge frequencies satisfy two. The node labeled by *ℓ*_3_ becomes a sub-parent, because both edge frequencies of different parents also meet two. Since *ℓ*_3_-list has no members with index zero, just two maximal frequent fractions can be derived, one is (*ℓ*_1_, {*ℓ*_1_,*ℓ*_3_}, {(*ℓ*_1_,*ℓ*_3_)}, *L*) (Recall that a tree *T* has a form of *T* = (*r, V, E, L*)) and the other (*ℓ*_2_, {*ℓ*_2_, *ℓ*_3_}, {(*ℓ*_2_, *ℓ*_3_)}, *L*).
- Case 2: *L*(*p*_1_) = *L*(*p*_2_) = *L*(*p*_3_) = *ℓ*_1_ and *L*(*p*_4_) = *ℓ*_2_. The edge (*ℓ*_1_,*ℓ*_3_) has the frequency three and, thus, satisfies the threshold two, but the edge (*ℓ*_2_, *ℓ*_3_) does not satisfies the threshold. As in Case 1, *ℓ*_3_-list has no members with index zero. Therefore, the number of maximal frequent fractions are still two, (*ℓ*_1_, {*ℓ*_1_, *ℓ*_3_}, {(*ℓ*_1_, *ℓ*_3_)}, *L*) and (*ℓ*_2_, {*ℓ*_2_}, {*ϕ*}, *L*).
- Case 3: *L*(*p*_1_) = *L*(*p*_2_) = 0 and *L*(*p*_3_) = *L*(*p*_4_) = *ℓ*_1_ or *ℓ*_2_*. ℓ*_3_-list has two members with index zero. According to the second condition of this case, we know that *ℓ*_1_ or *ℓ*_2_ is connected by *ℓ*_3_. Therefore, there are three maximal frequent fractions, (*ℓ*_1_ ||*ℓ*_2_, {*ℓ*_1_ || *ℓ*_2_}, {ϕ}, *L*), (*ℓ*_1_||*ℓ*_2_, {*ℓ*_1_||*ℓ*_2_, *ℓ*_3_}, {(*ℓ*_1_||*ℓ*_2_, *ℓ*_3_)}, *L*) and (*ℓ*_3_, {*ℓ*_3_}, {*ϕ*}, *L*).

Table 1 compares our mining scheme with two other schemes. As presented in the table, the three schemes are all based on the FP-Growth method of Han *et al.* [18]. However, Boukerche and Samarah's scheme [21] focuses on mining from simple relational stream data. Moreover, our scheme is the only one that considers the maximality of (t)items sets.

### 5.3. Correlating Concrete Contents with Label Lists

Let (*h_i_, b*_i_) and (*h_j_, b_j_*) be the head/body pairs of two arbitrary label lists *i*-list and *j*-list, respectively. Assume that the list sizes, |*i*-list| and |*j*-list|, are greater than |*XSD*| × *δ_s_.* Let *t_i_* and *t_j_* be the all tree indexes included in *b_i_* and *b_j_*, respectively. Then, the numbers of *t_i_* and *t_j_* are the same as the sizes of *i*-list and *j*-list, respectively. We denote a path between the two label lists by 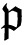
*_ij_* = (*h_i_, h_j_*), where *h_i_* is an ancestor of *h_j_* by Definition 3. Let 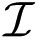 = {*I*_1_,…, *I_m_*} be a set of titems. Assume that *I*_1_ = 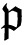
*_ij_* = (*h_i_, h_j_*) and *I*_2_ = 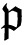
*_pq_* = (*h_p_, h_q_*) are two titems. Then, the confidence of *I*_1_ ⇒ *I*_2_ is computed as:

$$\text{conf}(I_{1}\Rightarrow I_{2},\text{XSD})=\frac{\text{freq}(I_{1}\cup I_{2},\text{XSD})}{\text{freq}(I_{1},\text{XSD})}=\frac{\left|t_{i}\cap t_{j}\cap t_{p}\cap t_{q}\right|}{\left|t_{i}\cap t_{j}\right|}.$$

#### Theorem 1

*Our proposed scheme extracts all of the XML stream data association rules for any dictionary* 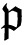
*_dic_ and for any values of sms and smc.*

##### proof

Let (*h_i_*, *b_i_*), (*h_j_*, *b_j_*) and (*h_k_*, *b_k_*) be three label lists in 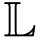
*_dic_*. Assume that |*t_i_* ∩ *t_k_*| = *β*, and |*t_i_* ∩ *t_j_* ∩ *t_k_*| = *γ*. Let 0 < *sms, smc* ≤ 1 and *x* and *y* be a tree index in *t_j_* and *t_k_*, respectively, such that *x* ≠ *y*.

titem*_ij_* (*x* ∈ *t_i_* Λ *y* ȩ *t_i_*): *h_i_* and *h_j_* forms 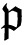
*_ij_*, which is a titem *I_ij_*. The support of titem*_ij_* is definitely greater than or equal to *sms*, due to the characteristics of 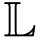
*_dic_.*
titem*_ik_* (*y* ∈ *t_i_* Λ *x* ȩ *t_i_*): *h_i_* and *h_k_* forms 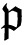
*_ik_*, which is a titem *I_ik_*. The support of titem*_ik_* is also greater than or equal to *sms.*
Association Rule: The confidence of an implication of the form *I_ij_* → *I_ik_* is computed by the following equation:
  $$\text{conf}(I_{ij}\Rightarrow I_{ik},\text{XSD})=\frac{\gamma }{\beta }.$$
  If *γ* ≥ *β* × *smc*, we have the rule *I_ij_* → *I_ik_* with the confidence greater than or equal to *smc*. Otherwise, we obtain the same rule *I_ij_* → *I_ik_*, but with the confidence less than *smc*.

## 6. Conclusion

This paper has introduced a comprehensive scheme for mining association rules from XML stream data. Our proposed scheme consists of a reformulation of association rules for XML streamed data, an extraction methodology both for individual blocks and the entire stream and a list-based structure for storing XML tree labels. Our scheme is unique in that it uses a list-based structure to deal with XML stream data. We showed that the FP-Growth-based structure combined with the label projected database can dramatically reduce the size of stream data, from 100% to 37.5% in our example, with respect to its units and frequent label lists. One of the advantages of our scheme is to achieve its goal without any redundancy in generating frequent tree items. Our scheme is also unique in that it uses and generates a maximal fraction that includes all frequent titems from XML stream data. Future work includes presenting a concrete mining scheme or algorithm that is proven to be correct and carries various experimental results for demonstrating high efficiency in different parameter settings.

## Figures and Tables

**Table 1.** A comparison of the characteristics.

**Scheme**	**Data**	**Base Approach**	**Maximality**
Corpinar and Gündem's scheme [10]	XML data	FP-Growth	No
Boukerche and Samarah's scheme [21]	Simple relational data	FP-Growth	No
Our scheme	XML data	FP-Growth	Yes
